# Carnivore chaphamaparvovirus-1 (CaChPV-1) infection in diarrheic dogs reveals viral endotheliotropism in intestine and lung

**DOI:** 10.1080/01652176.2023.2185696

**Published:** 2023-03-08

**Authors:** Chutchai Piewbang, Pattiya Lohavicharn, Tin Van Nguyen, Panitnan Punyathi, Tanit Kasantikul, Somporn Techangamsuwan

**Affiliations:** a Department of Pathology, Faculty of Veterinary Science, Chulalongkorn University, Bangkok, Thailand; b Animal Virome and Diagnostic Development Research Unit, Faculty of Veterinary Science, Chulalongkorn University, Bangkok, Thailand; c The International Graduate Course of Veterinary Science and Technology (VST), Faculty of Veterinary Science, Chulalongkorn University, Bangkok, Thailand; d Clemson Veterinary Diagnostic Center, Clemson University, Columbia, South Carolina, USA

**Keywords:** Canine, dog, Chaphamaparvovirus, CaChPV-1, cachavirus, parvovirus, diarrhea, enteritis, pneumonia

## Abstract

**Background:**

Carnivore chaphamaparvovirus-1 (CaChPV-1) is a parvovirus identified in dogs and association of infection with diarrhea is controversial. Information on whether tissue tropism persists is lacking.

**Objectives:**

To determine the disease association of CaChPV-1 in dogs with diarrhea and to investigate viral tropism and genetic diversity.

**Animals and methods:**

CaChPV-1 infection was investigated in five recently deceased puppies and designed a retrospective study to determine whether the presence of CaChPV-1 is associated with diarrhea. The retrospective study was conducted in 137 intestinal tissue samples and 168 fecal samples obtained from 305 dogs. CaChPV-1 tissue localization was determined using *in situ* hybridization, and CaChPV-1 complete genomes obtained from dead puppies and retrospective study were sequenced and analyzed.

**Results:**

CaChPV-1 was detected in 6.56% (20/305) of tested dogs, including 14 diarrheic- and 6 non-diarrheic dogs, and was significant in puppies with diarrhea (*p* = 0.048). Among the CaChPV-1-positive diarrheic dogs, one sample was obtained from intestinal tissue and 13 samples were fecal samples. However, six CaChPV-1 positive non-diarrheic dogs were based on fecal samples but not on intestinal tissue. Within the age range, the presence of CaChPV-1 was significant in puppies (*p* < 0.00001) and was mainly localized in the stromal and endothelial cells of intestinal villi and pulmonary alveoli. Phylogenetic analysis indicated genetic diversity of CaChPV-1 Thai strains that were mostly clustered within the sequences found in China.

**Conclusions:**

Although definitive pathogenesis of CaChPV-1 remains undetermined, this study provides evidence supporting that CaChPV-1 localizes in canine cells and could play a potential role as an enteric pathogen.

## 1. Introduction

Parvoviruses are non-enveloped, icosahedral viruses containing a negative sense, single-stranded DNA genome of 3.9–6.3 kb length (Berns and Parrish 2013). According to the current update by the International Committee on Taxonomy of Viruses (ICTV), parvoviruses belong to the *Parvoviridae* family, which presently includes *Parvovirinae*, *Densovirinae*, and novel *Hamaparvovirinae* subfamilies (Pénzes et al. 2020). The *Parvovirinae* and *Densovirinae* subfamilies are primarily distinguishable by their ability to infect only vertebrate and non-vertebrate hosts, respectively (Cotmore et al. 2019). Although the *Hamaparvovirinae* subfamily contains many viral genera that infect non-vertebrate hosts, many viruses belonging to the *Chaphamaparvovirus* genus have been reported to infect vertebrate hosts (Pénzes et al. 2020).

Regarding infection in dogs, there are several parvoviruses considered to be associated with diseases that mostly affect gastrointestinal systems (Jager et al. 2021). The canine parvovirus, formerly known as canine minute virus (CnMV) or canine parvovirus type 1 (CPV-1) and currently known as canine bocavirus-1 (CBoV-1; genus *Bocaparvovirus*), was first identified in the late nineteenth century, and it is currently known to be responsible for enteric and respiratory diseases in puppies and young dogs (Carmichael et al. 1994; Ohshima et al. 2010). A second canine parvovirus, or CPV-2 (genus *Protoparvovirus*), was discovered in dogs with severe hemorrhagic enteritis and myocarditis (Nho et al. 1997; Ford et al. 2017; Mazzaferro 2020). Since it has evolved, the original CPV2 has been displaced by its variants, including CPV-2a, CPV-2b, and CPV-2c, which are now regarded as major causative viruses for hemorrhagic enteritis of canines (Decaro and Buonavoglia 2012; Nguyen Manh et al. 2021). Furthermore, novel parvoviruses include 2 further species of canine bocaviruses, CBoV-2 and CBoV-3. The CBoV-2 is associated with respiratory and enteric disease in puppies (Bodewes et al. 2014; Piewbang et al. 2018). The CBoV-3 was discovered by deep-throughput molecular technology from the liver of a dog presenting multiorgan failures (Li et al. 2013). Subsequently, a novel parvovirus namely canine bufavirus (CBuF) was identified in fecal samples obtained from dogs with and without diarrhea, and in respiratory samples obtained from dogs with respiratory disease (Martella et al. 2018). Within a few years, metagenomic analysis of fecal samples obtained from dogs identified novel parvoviruses, initially named cachavirus-1A and -1B, that had a genetic relationship to viruses in the *Chaphamaparvovirus* (ChPV) genus (Fahsbender et al. 2019). Regarding this, the detected chaphamaparvovirus in dogs is classified as *carnivore chaphamaparvovirus-1* (CaChPV-1) according to an ICVT report in 2021 (Pénzes et al. 2019; Palombieri et al. 2020).

Although a variety of ChPVs have been identified in various hosts, including murine (Yang et al. 2016; Williams et al. 2018), bat (Souza et al. 2017; Yinda et al. 2018), pig (Palinski et al. 2016), birds (Wang et al. 2019), macaque (Kapusinszky et al. 2017), Tasmanian devil (Chong et al. 2019), peafowl (Liu et al. 2020), and turkey (Reuter et al. 2014), reports of CaChPV-1 infection in dogs have been relatively limited. There have been some anecdotal speculations of CaChPV-1 association with enteric disease in dogs (Fahsbender et al. 2019), although such an association was not demonstrated and not supported by statistical analysis (Hu et al. 2020; Palombieri et al. 2020). Considering the difficulties in elucidating the viral epidemiology and ability to cause disease, investigation on pathology-associated CaChPV-1 infection is essential to establish the possible role of CaChPV-1 in canine health. To obtain a detailed understanding of the disease association of CaChPV-1 in dogs, this study describes a concurrent CaChPV-1 infection in association with fatal enteric disease in a colony of puppies in Thailand. Association of pathological findings and presence of CaChPV-1 was also investigated. Retrospective investigation of CaChPV-1 in samples derived from two large cohorts obtained from deceased and living dogs with and without diarrhea was also performed.

## 2. Materials and methods

### 2.1. Fatal enteric disease outbreak and necropsy

Fatal enteric disease occurred in a French bulldog breeding colony in Bangkok, Thailand, in April 2021. A total of five 4-day-old puppies (dog nos.1–5) naturally delivered by a 1-year-old, CPV-2- and canine distemper (CDV)-vaccinated indoor dam developed progressive anorexia, bloody diarrhea and respiratory distress. All 5 puppies died within 2 days of presenting clinical signs. The dam presented with mild watery diarrhea a week before parturition with self-limiting clinical signs. All dead puppies were submitted for necropsy at the Department of Pathology, Faculty of Veterinary Science, Chulalongkorn University.

Fresh tissues, including heart, lung, liver, spleen, lymph nodes, intestines, kidneys, and bladder, were collected for molecular virologic screening including CPV (Piewbang et al. 2021a), CDV (Piewbang et al. 2020; Geiselhardt et al. 2022), canine herpesvirus-1 (CaHV-1) (Piewbang et al. 2017), canine adenoviruses (CAdVs) (Wardhani et al. 2021), canine enteric coronavirus (CCoV) (Ksiazek et al. 2003), canine bocavirus 1–3 (CBoV-1 to 3) (Piewbang et al. 2021b), canine bufavirus (CBuV) (Martella et al. 2018), canine astrovirus (CAstV) (Chu et al. 2008), and canine kobuvirus (CaKoV) (Li et al. 2018). Information regarding primers used for viral screening is described in Supplementary Table S1. Bacterial culture was performed from the lung and intestine samples. All collected fresh tissue samples were also immersed in 10% neutral buffered formalin for at least 48 h, embedded with paraffin, 4 µM thick sectioned, stained with hematoxylin and eosin (HE), and submitted for histological examination under a light microscope. Formalin-fixed, paraffin-embedded (FFPE) samples of lung and intestine were additionally subjected to periodic acid-Schiff (PAS) and phosphotungstic acid-hematoxylin (PTAH) staining.

**Table 1.** Correlation between CaChPV-1 detection and age of affected dogs.

CaChPV-1 PCR		Investigated dogs (%)^a^				
		Puppy	Young adult	Mature adult	Senior	Total
Positive		16 (5.25)^b^	2 (0.66)	1 (0.33)	1 (0.33)	20 (6.56)
	*Diarrheic* ^c^					
	Tissue	1	–	–	–	1
	Feces	11	1	–	1	13
	*Non-diarrheic*					
	Tissue	–	–	–	–	–
	Feces	4	1	1	–	6
Negative		64 (20.98)	77 (25.25)	68 (22.30)	76 (24.92)	285 (93.44)
	*Diarrheic*					
	Tissue	20	21	18	15	74
	Feces	8	11	17	26	62
	*Non-diarrheic*					
	Tissue	18	23	12	9	62
	Feces	18	22	21	26	87

^a^Puppy: birth to ≤9 months; young adult: >9 months to ≤4 years; mature adult: >4 years to ≤10 years; senior: >10 years.

^b^Statistical significance of CaChPV-1-positive detection among age group (Chi-square; *p* < 0.00001).

^c^Statistical significance of CaChPV-1-positive detection in diarrheic dogs (Chi-square; *p* = 0.048).

### 2.2. Detection of CaChPV-1 by conventional polymerase chain reaction (PCR)

The collected fresh tissue samples were individually minced and homogenized in 0.5% phosphate buffered saline (PBS) using an automatic homogenizer (Bead Ruptor 12, OMNI International, Kennesaw, GA, USA), and supernatant was collected. Viral DNA was extracted using the QIAmp MinElute Virus kit (Qiagen, Hilden, Germany). The quality and quantity of the extracted viral DNA were measured using a spectrophotometer at the A260/280 absorbent ratio (NanoDrop, Thermo Scientific™, Waltham, MA, USA). The 323-bp amplicon of partial nonstructural protein 1 (NS1) of the CaChV-1 was detected by nested PCR following the described protocol with minor modifications (Hu et al. 2020). PCR was performed using a thermocycler (BIO-RAD T100, Bio-Rad Laboratories, Hercules, CA, USA). Briefly, a total of 100 ng of extracted DNA was mixed with PCR kit reagents (2X GoTaq® Green Master Mix, Promega, Madison, WI, USA) containing 3 mM magnesium chloride, 400 µM deoxynucleotide triphosphate (dNTP) and *Taq* polymerase enzyme, and 10 pmol of each forward and reverse primer (F1: 5′CAACTAGCCGAATGCAGGGA3′ and R1: 5′CGATAACATCCCCGGACTGG3′). The cycling conditions were as follows: initial denaturation at 95 °C for 3 min, followed by 30 cycles of denaturation at 95 °C for 30 s, annealing at 55 °C for 30 s, and extension at 72 °C for 1 min, and final extension at 72 °C for 10 min. Negative controls were performed using non-template controls (NTC). The PCR products were visualized using the QIAxcel capillary electrophoresis platform (Qiagen, Hilden, Germany) with the setting described previously (Piewbang and Techangamsuwan 2019). Positive PCR products were subjected to sequencing using a next-generation sequencing (NGS)-based barcode-tagged sequencing platform (BTseq, U2BiO, Seoul, South Korea).

### 2.3. CaChPV-1 in situ hybridization (ISH)

FFPE tissues were sectioned, placed on a positively charged slide, and subsequently used for the CaChPV-1 chromogenic ISH assay. The CaChPV-1 DNA probe targeting 224 bp of the NS1 gene was constructed using a PCR DIG Probe Synthesis Kit (Roche Diagnostics, Basel, Switzerland). Briefly, the 224-bp DNA template of CaChPV-1 was obtained from a positive, sequencing-confirmed sample by PCR amplification using forward and reverse primers (F2: 5′AGCTCAGTTTGGCCCAGATC3′ and R2: 5′AGAGGGATCGCTGGATCTGT3′) with the following cycling conditions: initial denaturation at 95 °C for 3 min, followed by 30 cycles of denaturation at 95 °C for 30 s, annealing at 55 °C for 30 s, and extension at 72 °C for 1 min, and final extension at 72 °C for 10 min. The 224-bp PCR product was separated by running in 2% agarose gel electrophoresis, purified using a Monarch DNA Gel Extraction Kit (New England Biolab, Frankfurt, Germany), and subsequently used as a template for CaChPV-1 ISH probe construction. The thermal cycling and conditions were performed according to the PCR protocol described above, except for the use of digoxigenin (DIG)-labeled oligonucleotides. The constructed hybridization probe was confirmed by running on 2% (w/v) agarose gel electrophoresis in comparison with the PCR product derived from the use of normal, non-DIG-labeled oligonucleotides. Chromogenic ISH was performed according to a previously described protocol, except for the use of 55 °C overnight incubation. CaChPV-1 hybridization was detected by using a 1:200 anti-digoxigenin antibody (anti-DIG-AP Fab fragments, Roche, Basel, Switzerland) and a Liquid PermaRed/AP (Dako, Glostrup, Denmark) as the detection system. The CBoV-2 oligoprobe (Piewbang et al. 2021b) hybridization instead of ChPV-1 probe was performed as the negative control. A CPV-2-positive, CaChPV-1-negative intestinal section (Chaiyasak et al. 2020) and a CBoV-2-positive, CaChPV-1-negative intestinal section (Piewbang et al. 2021b) incubated with the CaChPV-1 probe were employed as additional negative controls. The slides were counterstained with Mayer’s hematoxylin and coverslipped. The positive hybridized signals were inspected under a light microscope, and the presence of red dot in ­association with cellular ­morphology was ­considered positive.

### 2.4. Retrospective study of CaChPV-1

A retrospective study was conducted by collecting two independent samples of (1) frozen intestinal tissues obtained from necropsied dogs that were routinely submitted for postmortem investigation at the Department of Pathology, Faculty of Veterinary Science, Chulalongkorn University, or (2) frozen fecal samples collected from living dogs that were patients at private animal hospitals/clinics in Bangkok, Thailand for various medical needs from January 2020 to June 2022. Two additional subsets in each sample group were further classified based on the presence or absence of observed clinical signs of gastroenteritis. Samples that showed positive detection in the routine virological tests as described above or samples that had a medical history of CPV or CCoV infection were excluded from this investigation. The studied dogs were classified into 4 age groups as puppy, young adult, mature adult, and senior according to the American Animal Hospital Association (AAHA) Canine Life Stage Guidelines 2019 (Creevy et al. 2019).

A total of 137 frozen intestinal samples retrieved from 75 diarrheic dogs and 62 non-diarrheic dogs, accompanied by a total of 168 fecal samples obtained from 75 diarrheic and 93 non-diarrheic dogs, were subjected for CaChPV-1 detection. Samples that presented positive-PCR detection were additionally selected and subsequently subjected to whole genome sequencing. The study was conducted in accordance with the ARRIVE guidelines and regulations and was approved by the Chulalongkorn University Animal Care and Use Committee (No. 2231006).

The Fisher exact test was used to determine the association of the presence of CaChPV-1 DNA in dogs with and without diarrhea. A chi-square test was used to determine the age range of affected dogs (Koletsi and Pandis 2016). The statistical analysis was performed on GraphPad, and *p* < 0.05 was considered statistically significant.

### 2.5. Whole genome sequencing and phylogenetic analysis of CaChPV-1

Representative CaChPV-1-positive DNA extracts including intestines from 5 necropsied puppies and fecal samples from 3 dogs were subjected to complete genome sequencing using multiple PCR assays following the described primers and thermal cycling protocols (Hu et al. 2020), with a variation of annealing temperature from 50 to 60 °C, optimized for each primer pair. The PCR product obtained in each reaction was submitted to NGS-based sequencing, as described above. The CaChPV-1 DNA sequences were aligned to a set of previous CaChPV-1 sequences and feline chaphamaparvovirus (FeChPV) sequences available in GenBank, and each sequence was mapped and assembled to construct the whole genome using BioEdit software version 7.0.5.3. Phylogenetic analysis based on complete coding sequences was performed using a maximum likelihood (ML) algorithm with a Hasegawa-Kishino-Yano with gamma distribution and invariable sites (HKY + G + I) model, following a find-best-fit model analysis based on the Bayesian information criterion (BIC) of nucleotide substitution and phylogenetically constructed with 1000 bootstrapping replicates embedded in the MEGA 11 software package (The Biodesign Institute, Tempe, AZ, USA). Phylogenetic analysis of NS1 and VP1 genes of CaChPV-1 was also performed using a similar algorithm, model, and settings to those described above. Pairwise nucleotide distance was measured to determine the genetic similarity to previously described CaChPV-1 sequences. The deduced amino acid sequences of obtained CaChPV-1 were compared with the sequences of previously reported CaChPV-1.

## 3. Results

### 3.1. Detection of CaChPV-1

CaChPV-1-nested PCR screening was positive in intestinal samples obtained from all dead puppies, with additional PCR-positive detection in the lungs and mesenteric lymph nodes of 3 puppies (nos. 2, 3, and 5). The CaChPV-1 DNA was also detected in the liver sample of puppy no. 3. The multiple PCR assays targeting canine enteric viruses and other common canine viruses were negative. *Escherichia coli* was isolated from intestinal samples, whereas no aerobic bacteria was cultivated from lung samples.

Investigation of CaChPV-1 in retrospective samples revealed positive detection in 0.73% (1/137) of intestinal samples from deceased dogs and 11.3% (19/168) of fecal samples from living dogs (*p* = 0.001). Among these CaChPV-1-positive samples, 70.0% (14/20) and 30.0% (6/20) were obtained from diarrheic and non-diarrheic dogs, respectively and they were not statistically significant among groups (*p* = 0.0651). Although it was not significantly different in CaChPV-1 detection between diarrheic and non-diarrheic dogs (determined from a total number of investigated dogs), the presence of CaChPV-1 DNA in fecal samples was statistically significant in living puppies showing diarrhea (*p* = 0.048). Furthermore, puppies also had the most significant positive detection rate (*p* < 0.00001) based on analysis among age group. Details regarding CaChPV-1 detection in different age groups are described in Table 1.

### 3.2. Genomic and phylogenetic analysis

**Figure 1.**
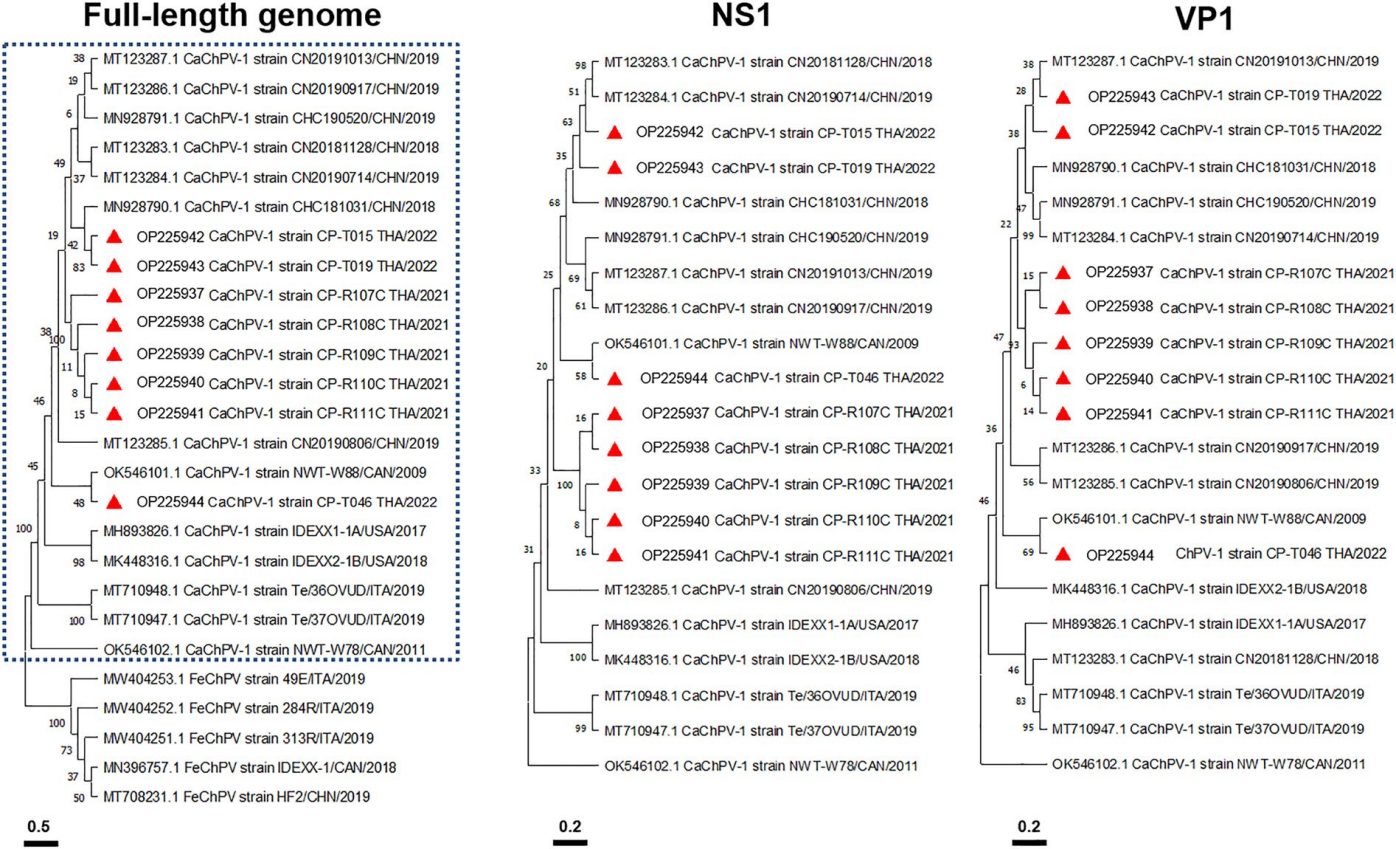
Phylogenetic topology of Carnivore chaphamaparvovirus-1 (CaChPV-1) based on full-length nucleotide sequences, non-structural gene 1 (NS1), and viral capsid gene 1 (VP1). The CaChPV-1 obtained from dogs presented a monophyletic clade (blue dot box) separating it from feline chaphamaparvovirus. The CaChPV-1 sequences obtained in this study (red triangles) were separately grouped into two lineages that were clustered with the CaChPV-1 sequences found in China and Canada, supported by phylogenetic analysis of the NS1 and VP1 genes. Bars indicate nucleotide substitution per site.

Eight full-length CaChPV-1 genomes obtained from 5 dead puppies (CaChPV-1 strains CP-R107–111C THA/2021), 2 diarrheic dogs (CaChPV-1 strains CP-T015 THA/2022 and CP-T019 THA/2022), and one non-diarrheic dog (CaChPV-1 CP-T046 THA/2022) were successfully characterized, resulting in 3617 to 3625-bp genomes being recovered. The obtained genomes sequences of CaChPV-1 strains CP-R107–111C THA/2021 and CP-T105, CP-T019, and CP-T046 THA/2022, were submitted to the NCBI database under the GenBank accession nos. OP225937–OP225944, respectively. The obtained CaChPV-1 genomes contained 3 main open reading frames (ORFs), ORFs 1–3, encoding putative nonstructural protein (NS), NS1, and viral capsid protein 1 (VP1), which presented the most variable nucleotide sequences among CaChPV-1 genomes. Pairwise nucleotide distances revealed that the CaChPV-1 sequences obtained from all necropsied puppies had genetic identical among sequences and diverged from the other CaChPV-1 sequences discovered from fecal samples of dogs in this study and other previously published CaChPV-1 sequences, accounting for 0.004–0.005% and 0.004–0.051%, respectively. Concordant with pairwise nucleotide analysis results, phylogenetic analysis of CaChPV-1 based on complete genome sequencing revealed that the obtained CaChPV-1 sequences were divergent, in which they were separately clustered into two lineages that were clustered with the CaChPV-1 sequences found in China and Canada (Figure 1). Most of the CaChPV-1 sequences found in this study were genetically close to the sequences found in China, except the CaChPV-1 CP-T046 THA/2022 strain, which clustered with the CaChPV-1 NWT-W88/CAN/2019 found in Canada. Interestingly, the CaChPV-1 sequences discovered from deceased puppies clustered together and created a unique phylogenetic clade close to the CaChPV-1 sequences found in China. Phylogenetic analysis of NS1 and VP1 nucleotide sequences supported the findings by presenting a unique phylogenic topology of the CaChPV-1 obtained from deceased puppies. However, deduced amino acid changes found in the CaChPV-1 sequences obtaining from the dead puppies were only present in the NS1 region, which included M104L, D601N, and A631T.

### 3.3. Gross and histological findings

All 5 puppies had relatively similar gross lesions. The cadavers were in normal body condition with evidence of yellowish watery diarrhea. The lungs of all investigated puppies were red and consolidated. Gastrointestinal examination revealed diffuse catarrhal, foul-smelling content along with the intestine, together with enlarged and congested mesenteric lymph nodes. Petechial hemorrhages were also found on all footpads of one examined puppy (case no. 2). Severe hepatic congestion was seen in case nos. 2 and 3.

**Figure 2.**
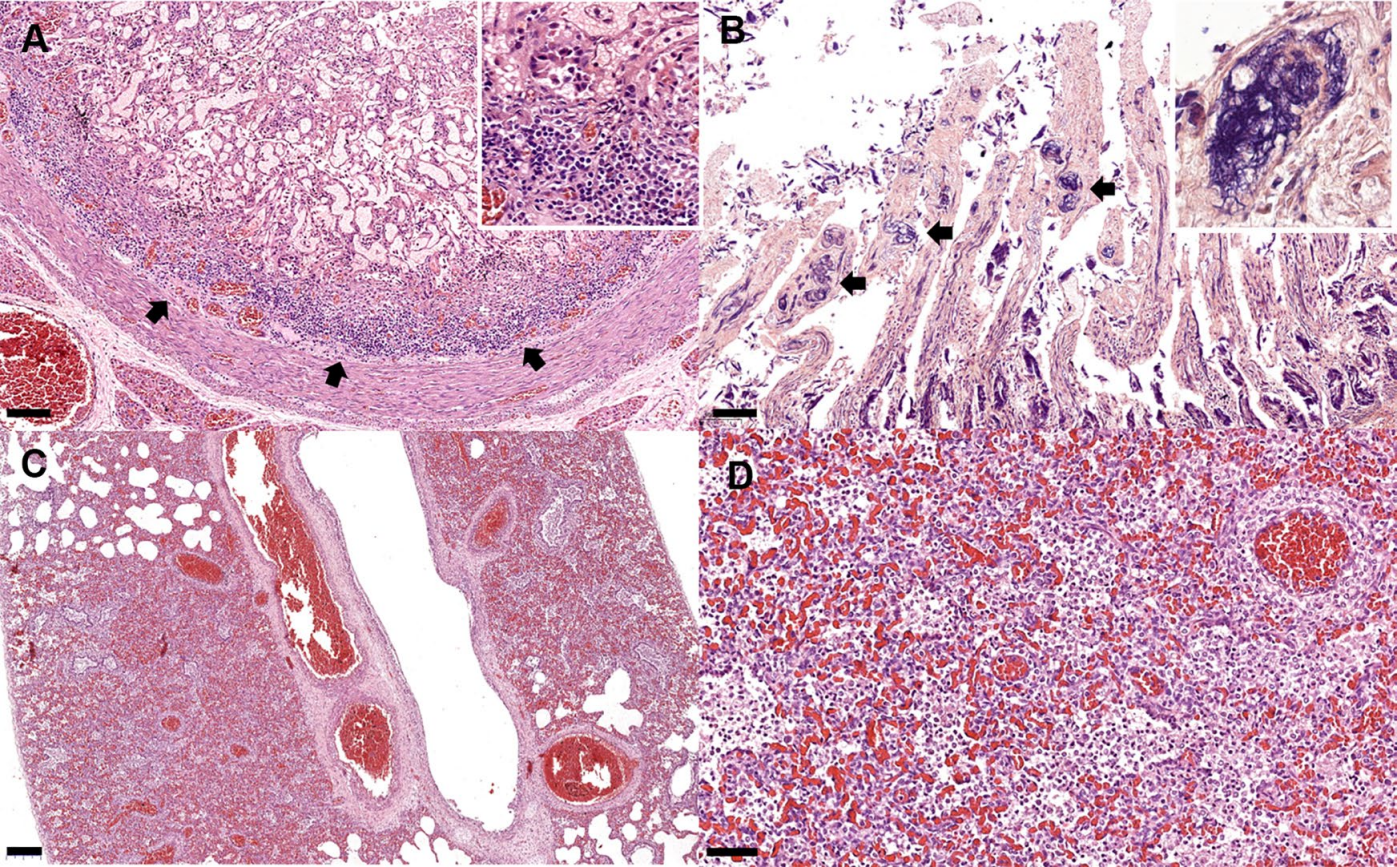
Carnivore chaphamaparvovirus-1 infection. Puppy no. 2. Duodenum (A&B). (A) Focally extensive lymphoplasmacytic duodenitis (arrows) and villous necrosis. Lymphocyte and plasma cell infiltration in intestinal submucosa (inset). Hematoxylin & eosin (H&E) staining. (B) The lumen of several blood vessels within the remnant villous structures are filled with granular to fibrillar materials (arrows), highlighted by deep blue color (inset). Phosphotungstic acid-hematoxylin (PTAH) staining. Puppy no. 3. Lung (C&B). (C) Interstitial pneumonia and diffuse pulmonary congestion. The alveolar lumen of small bronchiolar airways is filled with pools of inflammatory cells, and lining type II pneumocytes segmentally exhibit tombstoning hypertrophy. (D) These inflammatory cells are predominantly composed of lymphocytes, neutrophils, and foamy macrophages. H&E. Bars indicate 100 µm (A&B), 200 µm (C), and 50 µm (D).

Histologically, small intestinal sections exhibited moderate focally extensive lymphoplasmacytic enteritis and villous necrosis (Figure 2A). Variable degrees of enteritis were present throughout the small intestine, in which the duodenum was the most affected region. There were variable infiltrates of lymphocytes and plasma cells noted within the remaining intestinal mucosa. Crypt epithelial cells varied from attenuated to plump and tall columnar with large vesiculated nuclei containing large basophilic nucleoli. A few crypt epithelial cells exhibited mitotic figures. Occasionally crypts were often filled with eosinophilic and cellular debris. The lumen of several blood vessels within the remnant villous structures was filled with eosinophilic granular to fibrillar material. There were scant laminated mats of fibrin within the lumen of occasional small blood vessels, highlighted by PTAH staining (Figure 2B).

The lumen of alveolar spaces, bronchioles and terminal airways were filled with variable aggregates of foamy macrophages and neutrophils intermixed with scant eosinophilic granular material (Figure 2C). Pulmonary alveoli were multifocally collapsed and diffusely variably hypercellular with associated infiltrates of histiocytes, lymphocytes, and fewer neutrophils and increased prominence of engorged and tortuous alveolar capillaries (Figure 2D). Many macrophages containing black pigment globules were scattered throughout the lung. There were no remarkable lesions found in other organs, histologically.

### 3.4. PCR and ISH

**Figure 3.**
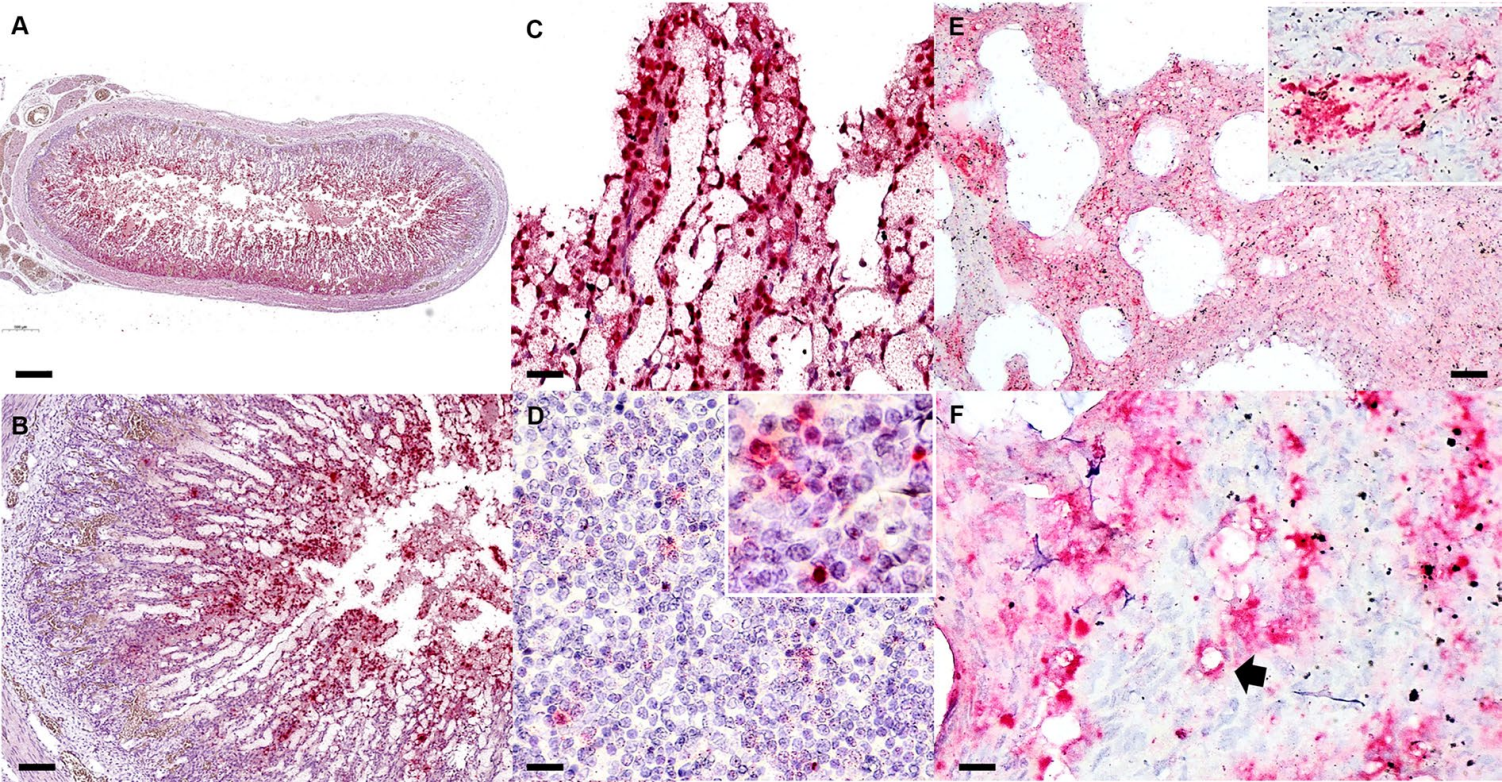
Carnivore chaphamaparvovirus-1 infection. *In situ* hybridization. Puppy no. 2. Intestine (A-C). Mesenteric lymph node (D). Lung (E&F). (A) Large amounts of carnivore chaphamarparvovirus-1 (CaChPV-1) nucleic acid in the duodenum. (B, C) The CaChPV-1 DNA is present in the superficial mucosa of intestinal villi. The CaChPV-1 nucleic acid is primarily detected within the nuclei of supporting mesenchymal cells and endothelial cells of villous capillaries. (D) CaChPV-1 DNA is detected in single lymphocytes and histiocytes in the mesenteric lymph node. (E) Large amounts of CaChPV-1 nucleic acid are multifocally present in pulmonary parenchyma and small blood vessels (inset). (F) CaChPV-1 hybridization signals are primarily present in the nuclei of infiltrated histiocytes and lymphocytes in alveoli and present in lining endothelial cells of alveolar capillaries (arrow). Bars indicate 500 µm (A), 100 µm (B&E), 20 µm (C&F), and 10 µm (D).

PCR analysis of fresh tissue samples detected CaChPV-1 DNA in intestinal samples of all investigated puppies, with additional CaChPV-1 DNA detection in the mesenteric lymph node and lung of case nos. 2 and 3. The CaChPV-1 DNA was also detected in the liver of case no. 2 by PCR (Table 2). ISH identified a large amount of CaChPV-1 nucleic acid within small intestinal tissues (Figure 3A). Viral nucleic acid was primarily detected within the nuclei of supporting mesenchymal cells (Figure 3B) and lining endothelial cells of villous capillaries of the superficial mucosal intestinal villi (Figure 3C). In the other regions of the small intestine, viral nucleic acid was detected in the nuclei of rare, scattered individual cells such as supporting stromal cells. Only a few nuclei of regenerating crypt epithelial cells labelled positive by ISH for CaChPV-1 DNA were also identified. Rare, intranuclear CaChPV-1 hybridization signals were observed in inflammatory cells in the mesenteric lymph node sections of case no. 2 (Figure 3D). Additionally, the CaChPV-1 DNA was identified in the bronchial epithelium and lining endothelial cells of pulmonary alveoli (Figure 3E) and small blood vessels of case nos. 2 and 3 (Figure 3F). All other organs examined were negative for CaChPV-1 by ISH. No hybridization signal presented in the negative controls. Details regarding CaChPV-1 detection are described in Table 2.

**Table 2.** Detection of CaChPV-1 in various organs of necropsied canine puppies with fatal enteric disease.

Animal	Sample	Detection method	
		PCR	ISH
1	Intestine	+	+
	MLN	–	–
	Others^a^	–	–
2	Intestine	+	+
	MLN	+	+
	Lung	+	+
	Liver	+	–
	Others^a^	–	–
3	Intestine	+	+
	MLN	+	–
	Lung	+	+
	Others^a^	–	–
4	Intestine	+	+
	MLN	–	–
	Others^a^	–	–
5	Intestine	+	NA
	MLN	–	NA
	Others^a^	–	NA

ISH, *in situ* hybridization; MLN, mesenteric lymph node; NA, not perform; PCR, polymerase chain reaction.

^a^Other organs including heart, liver, spleen, kidneys, and bladder.

## 4. Discussion

Although the presence of CaChPV-1 DNA in cases of canine enteric diseases has been reported (Fahsbender et al. 2019; Hu et al. 2020; Palombieri et al. 2020), information regarding CaChPV-1 tropism nor viral distribution in the intestine or other organs, is established. So far, the association of CaChPV-1 with canine enteric diseases remains controversial. In this study, we initially identified CaChPV-1 in association with a fatal enteric disease outbreak, prompting us to further investigate large numbers of two retrospective cohorts deriving from both deceased and living dogs that had clinical signs with and without diarrhea.

The findings of this study – although definitive pathogenesis of CaChPV-1 causing enteric disease and conclusive results indicate this virus served as an etiological agent causing fatal disease remain to be determined – could provide strong evidence supporting that CaChPV-1 might play a potential role as an enteric pathogen of canines, as shown by statistical analysis and evidence of CaChPV-1 identification in diarrheic dogs without concomitant infection. Previous studies found that CaChPV-1-positive dogs with diarrhea were coinfected with other canine viral enteric pathogens (Hu et al. 2020; Palombieri et al. 2020), potentially inhibiting interpretation of the exact role of CaChPV-1 alone that elicits the enteric disease in dogs. Regarding this limitation of previous studies, plus lacking *in vitro* or *in vivo* experiments, we designed this investigation by collecting retrospective samples from dogs that had not undergone detection of common enteric viruses in an attempt to indicate the possible role of CaChPV-1 in cases of diarrhea. We found that the presence of CaChPV-1 is not significant in dogs with diarrhea when determined from retrospectively investigated dogs. This finding is in accordance with previous research (Hu et al. 2020; Palombieri et al. 2020). However, when examining only samples obtained from living dogs, we observed the statistically significant finding of the presence of CaChPV-1 in puppies with diarrhea, similar to what was described in a previous study (Fahsbender et al. 2019). Specifically, investigation of 137 frozen intestinal tissues revealed only one sample showing CaChPV-1 positivity while the detection rate was higher in fecal samples obtained from living dogs suggesting mild clinical disease when infected. However, this speculation needs clarification in future research.

Interestingly, all CaChPV-1-positive deceased dogs were puppies, and analysis of age-related CaChPV-1 infection indicated significant correlation in the puppy age. These findings reflect that CaChPV-1 serves as a highly pathogenic virus for puppies but has less pathogenicity in adults as the presence of CaChV-1 was significantly observed in puppies with diarrhea when examining only the group of living dogs. Whether CaChPV-1 elicits enteric infection exacerbating severe clinical outcomes in puppies but mild to rare clinical course in adults should be definitively investigated. There are other infections or systemic diseases associated with diarrhea that could not be definitively ruled out in this study, even though we attempted to exclude some common canine enteric viral pathogens, so association of CaChPV-1 with canine enteric disease requires further observations.

As a specific cause of a fatal outbreak in breeding colony is unknown and was negative by routine virologic screening tests, we investigated the potential pathogens associated with such outbreak. We only detected CaChPV-1 but no other common canine viral pathogens in all deceased puppies, which might suggest that this virus served as a potential etiological agent associated with this fatal outbreak. The detection of this virus in all deceased puppies and the overlapping timeline of disease course suggest that this virus is contagious, as found in other parvovirus infections (Steinel et al. 2001; Decaro and Buonavoglia 2012; Chaiyasak et al. 2020; Piewbang et al. 2021a). However, samples obtained from the post-diarrheic dam and other neighboring animals were not available, so the origin of infection throughout infective sources could not be determined in this study.

Although CaChPV-1 is related to the parvovirus, the CaChPV-1 nucleic acid is detected in stromal or endothelial cells rather than crypt epithelial cells and enterocytes, as commonly found in CPV infection. CaChPV-1 localization in endothelial and supporting mesenchymal cells of villi suggests endotheliotropism of this virus (Bachelier et al. 2017; Piewbang et al. 2019, 2022). Corresponding with enteritis, identification of the virus in the lesion also supports the association with fatal enteric disease in puppies. Not only enteritis but also pulmonary diseases have been reported in a variety of parvovirus infections, such as the CnMV infection in puppies (Carmichael et al. 1994), bocavirus infection in pneumonic humans and animals (Akturk et al. 2015; Khalfaoui et al. 2016), and parvovirus B19 in cases of acute lung injury (Wardeh and Marik 1998; Ma et al. 2022). Apart from enteric disease, the CaChPV-1-positive puppies also showed respiratory distress which was supported by evidence of gross and histological lesions of the lung suggesting pneumonia. Identification of the CaChPV-1 localization in epithelial cells lining of pulmonary alveoli and endothelial cells of small blood vessels in the lungs of affected puppies without detection of concomitant viruses may indicate the possible role of the CaChPV-1 association with pneumonia. However, it is unclear regarding the cause of death of these puppies whether the virus contributed to endothelial cell damage resulting in fibrin thrombosis in blood vessels and capillaries and whether the subsequent systemic inflammatory reaction directly damaged the intestine and lung simultaneously. This disease requires further studies to provide a more fundamental understanding of pathogenesis. Although there is no information regarding CaChV-1 in association with canine respiratory disease, FeChPV, a genetically closely related CaChPV-1, was recently detected in cats with enteric and respiratory diseases (Di Profio et al. 2022), indicating the potential role of these chaphamaparvoviruses in both enteric and respiratory diseases. Studies focusing on CaChPV-1 in dogs with respiratory disease should not be overlooked. Furthermore, additional demonstration of CaChPV-1 in liver of the dead puppy no. 2 by PCR, in the absence of CaChPV-1 ISH signals, may indicate the hematogenous spread. Determination of the CaChPV-1 loads in various organs of infected animals may indicate whether viral tropism or hematogenous spread that would be interesting in future study.

Herein, we characterized the full-length genome of detected CaChPV-1 obtained from deceased diarrheic and healthy dogs to indicate the effect of genetic relatedness with clinical presentation. However, there are no genetic signatures among obtained CaChPV-1 sequences. This study also extended genetic analysis by comparison with previous studies on CaChPV-1 and found that the CaChPV-1 sequences obtained in this study were divergent and shared a close genetic relationship with the CaChPV-1 sequences in China, suggesting that these viruses have shared undefined origins. We also observed that the CaChPV-1 strain CP-T046 THA/2022 was genetically close to the CaChPV-1 found in Canada, which could indicate the genetic variation of the CaChPV-1 Thai strain. Similar to a recent study (Dinçer et al. 2020), we observed the deduced amino acid mutations encoded by the NS1 gene, which reflected differences between previously described CaChPV-1 strains and the strains found in this study. Although the NS1 protein is associated with viral pathogenicity of parvoviruses, the effects of these changes on CaChPV-1 remain to be determined and therefore require future studies.

Previous reports found that CaChPV-1 was often detected in cases of coinfection with other canine viral pathogens (Hu et al. 2020; Palombieri et al. 2020). This study investigated the presence of CaChPV-1 in dogs that had no concomitant viral infection, as our inclusion criteria for establishing the possible role of enteric disease; definitive epidemiological data of CaChPV-1 in dogs in Thailand could not be addressed.

In summary, this study provides novel information regarding CaChPV-1 in association with canine enteritis by establishing tissue localization of this virus in infected dogs. The findings also extend information on the CaChPV-1 distribution in Thailand throughout its genetic diversity, raising concern about potential pathogens affecting canine health.

## Supplementary Material

Supplemental Material

## Data Availability

All the data supporting our findings is contained within the manuscript. Eight full-length coding sequences of the CaChPV-1 have been deposited in NCBI GenBank under accession numbers OP225937–OP225944.

## References

[CIT0001] Akturk H, Sık G, Salman N, Sutcu M, Tatli B, Ciblak MA, Erol OB, Torun SH, Citak A, Somer A. 2015. Atypical presentation of human bocavirus: severe respiratory tract infection complicated with encephalopathy. J Med Virol. 87(11):1831–1838. 25966820 10.1002/jmv.24263 PMC7166798

[CIT0002] Bachelier K, Biehl S, Schwarz V, Kindermann I, Kandolf R, Sauter M, Ukena C, Yilmaz A, Sliwa K, Bock CT, et al. 2017. Parvovirus B19-induced vascular damage in the heart is associated with elevated circulating endothelial microparticles. PLoS One. 12(5):e0176311. 28531186 10.1371/journal.pone.0176311 PMC5439674

[CIT0003] Berns, K., & Parrish, C. (2013). Chapter 57: parvoviridae. In: Knipe DM, Howley PM, editors. Fields virology. Vols. 1 and 2. 6th ed. Philadelphia, PA: Lippincott Williams &Wilkins. p. 1768–1791.

[CIT0004] Bodewes R, Lapp S, Hahn K, Habierski A, Forster C, Konig M, Wohlsein P, Osterhaus AD, Baumgartner W. 2014. Novel canine bocavirus strain associated with severe enteritis in a dog litter. Vet Microbiol. 174(1–2):1–8. 25263495 10.1016/j.vetmic.2014.08.025 PMC7117162

[CIT0005] Carmichael LE, Schlafer DH, Hashimoto A. 1994. Minute virus of canines (MVC, canine parvovirus type-1): pathogenicity for pups and seroprevalence estimate. J Vet Diagn Invest. 6(2):165–174. 8068747 10.1177/104063879400600206

[CIT0006] Chaiyasak S, Piewbang C, Banlunara W, Techangamsuwan S. 2020. Carnivore protoparvovirus-1 associated with an outbreak of hemorrhagic gastroenteritis in small Indian civets. Vet Pathol. 57(5):706–713. 32880233 10.1177/0300985820932144

[CIT0007] Chong R, Shi M, Grueber CE, Holmes EC, Hogg CJ, Belov K, Barrs VR. 2019. Fecal viral diversity of captive and wild tasmanian devils characterized using virion-enriched metagenomics and metatranscriptomics. J Virol. 93(11):e00205–19. 30867308 10.1128/JVI.00205-19 PMC6532096

[CIT0008] Chu DK, Poon LL, Guan Y, Peiris JS. 2008. Novel astroviruses in insectivorous bats. J Virol. 82(18):9107–9114. 18550669 10.1128/JVI.00857-08 PMC2546893

[CIT0009] Cotmore SF, Agbandje-McKenna M, Canuti M, Chiorini JA, Eis-Hubinger AM, Hughes J, Mietzsch M, Modha S, Ogliastro M, Pénzes JJ, et al. 2019. ICTV virus taxonomy profile: parvoviridae. J Gen Virol. 100(3):367–368. 30672729 10.1099/jgv.0.001212 PMC6537627

[CIT0010] Creevy KE, Grady J, Little SE, Moore GE, Strickler BG, Thompson S, Webb JA. 2019. 2019 AAHA canine life stage guidelines. J Am Anim Hosp Assoc. 55(6):267–290. 31622127 10.5326/JAAHA-MS-6999

[CIT0011] Decaro N, Buonavoglia C. 2012. Canine parvovirus – a review of epidemiological and diagnostic aspects, with emphasis on type 2c. Vet Microbiol. 155(1):1–12. 21962408 10.1016/j.vetmic.2011.09.007 PMC7173204

[CIT0012] Di Profio F, Sarchese V, Palombieri A, Fruci P, Massirio I, Martella V, Fulvio M, Di Martino B. 2022. Feline chaphamaparvovirus in cats with enteritis and upper respiratory tract disease. Transbound Emerg Dis. 69(2):660–668. 33559350 10.1111/tbed.14032

[CIT0013] Dinçer E, Timurkan MÖ, Dinçer PFP, Aydın H. 2020. Co-circulation of canine chaphamaparvovirus and canine parvovirus 2 in dogs with diarrhea in Turkey. Thai J Vet Med. 50(4):495–501. https://he01.tci-thaijo.org/index.php/tjvm/article/view/246317.

[CIT0014] Fahsbender E, Altan E, Seguin MA, Young P, Estrada M, Leutenegger C, Delwart E. 2019. Chapparvovirus DNA found in 4% of dogs with diarrhea. Viruses. 11(5):398. 31035625 10.3390/v11050398 PMC6563200

[CIT0015] Ford J, McEndaffer L, Renshaw R, Molesan A, Kelly K. 2017. Parvovirus infection is associated with myocarditis and myocardial fibrosis in young dogs. Vet Pathol. 54(6):964–971. 28812526 10.1177/0300985817725387 PMC10984720

[CIT0016] Geiselhardt F, Peters M, Jo WK, Schadenhofer A, Puff C, Baumgärtner W, Kydyrmanov A, Kuiken T, Piewbang C, Techangamsuwan S, et al. 2022. Development and validation of a pan-genotypic real-time quantitative reverse transcription-PCR assay to detect canine distemper virus and phocine distemper virus in domestic animals and wildlife. J Clin Microbiol. 60(5):e0250521. 35491822 10.1128/jcm.02505-21 PMC9116185

[CIT0017] Hu W, Liu Q, Chen Q, Ji J. 2020. Molecular characterization of Cachavirus firstly detected in dogs in China. Infect Genet Evol. 85:104529. 32890765 10.1016/j.meegid.2020.104529 PMC7468343

[CIT0018] Jager MC, Tomlinson JE, Lopez-Astacio RA, Parrish CR, Van de Walle GR. 2021. Small but mighty: old and new parvoviruses of veterinary significance. Virol J. 18(1):210. 34689822 10.1186/s12985-021-01677-y PMC8542416

[CIT0019] Kapusinszky B, Ardeshir A, Mulvaney U, Deng X, Delwart E. 2017. Case-control comparison of enteric viromes in captive rhesus macaques with acute or idiopathic chronic diarrhea. J Virol. 91(18):e00952–17. 28659484 10.1128/JVI.00952-17 PMC5571273

[CIT0020] Khalfaoui S, Eichhorn V, Karagiannidis C, Bayh I, Brockmann M, Pieper M, Windisch W, Schildgen O, Schildgen V. 2016. Lung infection by human bocavirus induces the release of profibrotic mediator cytokines in vivo and in vitro. PLoS One. 11(1):e0147010. 26807786 10.1371/journal.pone.0147010 PMC4726461

[CIT0021] Koletsi D, Pandis N. 2016. The chi-square test for trend. Am J Orthod Dentofacial Orthop. 150(6):1066–1067. 27894529 10.1016/j.ajodo.2016.10.001

[CIT0022] Ksiazek TG, Erdman D, Goldsmith CS, Zaki SR, Peret T, Emery S, Tong S, Urbani C, Comer JA, Lim W, SARS Working Group, et al. 2003. A novel coronavirus associated with severe acute respiratory syndrome. N Engl J Med. 348(20):1953–1966. 12690092 10.1056/NEJMoa030781

[CIT0023] Li L, Pesavento PA, Leutenegger CM, Estrada M, Coffey LL, Naccache SN, Samayoa E, Chiu C, Qiu J, Wang C, et al. 2013. A novel bocavirus in canine liver. Virol J. 10(1):54. 23402347 10.1186/1743-422X-10-54 PMC3577433

[CIT0024] Li M, Yan N, Wang M, Zhang B, Yue H, Tang C. 2018. Prevalence and genomic characteristics of canine kobuvirus in southwest China. Arch Virol. 163(2):459–466. 29143141 10.1007/s00705-017-3648-y

[CIT0025] Liu X, Wang H, Liu X, Li Y, Chen J, Zhang J, Wang X, Shen S, Wang H, Deng F, et al. 2020. Genomic and transcriptional analyses of novel parvoviruses identified from dead peafowl. Virology. 539:80–91. 31706163 10.1016/j.virol.2019.10.013

[CIT0026] Ma M, Ma X, Jia M, Hou X, Wang H. 2022. Adult acute respiratory distress syndrome due to human parvovirus B19 infection after cardiac surgery: a case report. BMC Infect Dis. 22(1):231. 35255838 10.1186/s12879-022-07213-9 PMC8899786

[CIT0027] Martella V, Lanave G, Mihalov-Kovács E, Marton S, Varga-Kugler R, Kaszab E, Di Martino B, Camero M, Decaro N, Buonavoglia C, et al. 2018. Novel parvovirus related to primate bufaviruses in dogs. Emerg Infect Dis. 24(6):1061–1068. 29774829 10.3201/eid2406.171965 PMC6004837

[CIT0028] Mazzaferro EM. 2020. Update on canine parvoviral enteritis. Vet Clin North Am Small Anim Pract. 50(6):1307–1325. 32891439 10.1016/j.cvsm.2020.07.008 PMC7467068

[CIT0029] Nguyen Manh T, Piewbang C, Rungsipipat A, Techangamsuwan S. 2021. Molecular and phylogenetic analysis of Vietnamese canine parvovirus 2C originated from dogs reveals a new Asia-IV clade. Transbound Emerg Dis. 68(3):1445–1453. 32854156 10.1111/tbed.13811

[CIT0030] Nho WG, Sur JH, Doster AR, Kim SB. 1997. Detection of canine parvovirus in naturally infected dogs with enteritis and myocarditis by in situ hybridization. J Vet Diagn Invest. 9(3):255–260. 9249164 10.1177/104063879700900306

[CIT0031] Ohshima T, Kawakami K, Abe T, Mochizuki M. 2010. A minute virus of canines (MVC: canine bocavirus) isolated from an elderly dog with severe gastroenteritis, and phylogenetic analysis of MVC strains. Vet Microbiol. 145(3–4):334–338. 20427134 10.1016/j.vetmic.2010.03.033 PMC7117362

[CIT0032] Palinski RM, Mitra N, Hause BM. 2016. Discovery of a novel Parvovirinae virus, porcine parvovirus 7, by metagenomic sequencing of porcine rectal swabs. Virus Genes. 52(4):564–567. 26995221 10.1007/s11262-016-1322-1

[CIT0033] Palombieri A, Di Profio F, Lanave G, Capozza P, Marsilio F, Martella V, Di Martino B. 2020. Molecular detection and characterization of Carnivore chaphamaparvovirus 1 in dogs. Vet Microbiol. 251:108878. 33069035 10.1016/j.vetmic.2020.108878 PMC7528909

[CIT0034] Pénzes JJ, de Souza WM, Agbandje-McKenna M, Gifford RJ. 2019. An ancient lineage of highly divergent parvoviruses infects both vertebrate and invertebrate hosts. Viruses. 11(6):525. 31174309 10.3390/v11060525 PMC6631224

[CIT0035] Pénzes JJ, Söderlund-Venermo M, Canuti M, Eis-Hübinger AM, Hughes J, Cotmore SF, Harrach B. 2020. Reorganizing the family Parvoviridae: a revised taxonomy independent of the canonical approach based on host association. Arch Virol. 165(9):2133–2146. 32533329 10.1007/s00705-020-04632-4

[CIT0036] Piewbang C, Techangamsuwan S. 2019. Phylogenetic evidence of a novel lineage of canine pneumovirus and a naturally recombinant strain isolated from dogs with respiratory illness in Thailand. BMC Vet Res. 15(1):300. 31426794 10.1186/s12917-019-2035-1 PMC6700830

[CIT0037] Piewbang C, Rungsipipat A, Poovorawan Y, Techangamsuwan S. 2017. Viral molecular and pathological investigations of Canid herpesvirus 1 infection associated respiratory disease and acute death in dogs. Acta Vet Brno. 67(1):11–24.

[CIT0038] Piewbang C, Jo WK, Puff C, Ludlow M, van der Vries E, Banlunara W, Rungsipipat A, Kruppa J, Jung K, Techangamsuwan S, et al. 2018. Canine bocavirus type 2 infection associated with intestinal lesions. Vet Pathol. 55(3):434–441. 29421972 10.1177/0300985818755253

[CIT0039] Piewbang C, Kasantikul T, Pringproa K, Techangamsuwan S. 2019. Feline bocavirus-1 associated with outbreaks of hemorrhagic enteritis in household cats: potential first evidence of a pathological role, viral tropism and natural genetic recombination. Sci Rep. 9(1):16367. 31705016 10.1038/s41598-019-52902-2 PMC6841677

[CIT0040] Piewbang C, Chansaenroj J, Kongmakee P, Banlunara W, Poovorawan Y, Techangamsuwan S. 2020. Genetic adaptations, biases, and evolutionary analysis of canine distemper virus Asia-4 lineage in a fatal outbreak of wild-caught civets in Thailand. Viruses. 12(4):361. 32224857 10.3390/v12040361 PMC7232145

[CIT0041] Piewbang C, Wardhani SW, Chanseanroj J, Yostawonkul J, Boonrungsiman S, Saengkrit N, Kongmakee P, Banlunara W, Poovorawan Y, Kasantikul T, et al. 2021a. Natural infection of parvovirus in wild fishing cats (Prionailurus viverrinus) reveals extant viral localization in kidneys. PLoS One. 16(3):e0247266. 33651823 10.1371/journal.pone.0247266 PMC7924760

[CIT0042] Piewbang C, Wardhani SW, Dankaona W, Lacharoje S, Chai-In P, Yostawonkul J, Chanseanroj J, Boonrungsiman S, Kasantikul T, Poovorawan Y, et al. 2021b. Canine bocavirus-2 infection and its possible association with encephalopathy in domestic dogs. PLoS One. 16(8):e0255425. 34383794 10.1371/journal.pone.0255425 PMC8360608

[CIT0043] Piewbang C, Wardhani SW, Phongroop K, Lohavicharn P, Sirivisoot S, Kasantikul T, Techangamsuwan S. 2022. Naturally acquired feline bocavirus type 1 and 3 infections in cats with neurologic deficits. Transbound Emerg Dis. 69(5):e3076–e3087. 35869862 10.1111/tbed.14664

[CIT0044] Reuter G, Boros Á, Delwart E, Pankovics P. 2014. Novel circular single-stranded DNA virus from turkey faeces. Arch Virol. 159(8):2161–2164. 24562429 10.1007/s00705-014-2025-3

[CIT0045] Souza WM, Romeiro MF, Fumagalli MJ, Modha S, de Araujo J, Queiroz LH, Durigon EL, Figueiredo LTM, Murcia PR, Gifford RJ. 2017. Chapparvoviruses occur in at least three vertebrate classes and have a broad biogeographic distribution. J Gen Virol. 98(2):225–229. 28284244 10.1099/jgv.0.000671 PMC5646239

[CIT0046] Steinel A, Parrish CR, Bloom ME, Truyen U. 2001. Parvovirus infections in wild carnivores. J Wildl Dis. 37(3):594–607. 11504234 10.7589/0090-3558-37.3.594

[CIT0047] Wang Y, Yang S, Liu D, Zhou C, Li W, Lin Y, Wang X, Shen Q, Wang H, Li C, et al. 2019. The fecal virome of red-crowned cranes. Arch Virol. 164(1):3–16. 30225519 10.1007/s00705-018-4037-x PMC7086969

[CIT0048] Wardeh A, Marik P. 1998. Acute lung injury due to parvovirus pneumonia. J Intern Med. 244(3):257–260. 9747749 10.1046/j.1365-2796.1998.00364.x

[CIT0049] Wardhani SW, Wongsakul B, Kasantikul T, Piewbang C, Techangamsuwan S. 2021. Molecular and pathological investigations of selected viral neuropathogens in rabies-negative brains of cats and dogs revealed neurotropism of carnivore protoparvovirus-1. Front Vet Sc. 8(937):710701. 34490401 10.3389/fvets.2021.710701 PMC8416986

[CIT0050] Williams SH, Che X, Garcia JA, Klena JD, Lee B, Muller D, Ulrich W, Corrigan RM, Nichol S, Jain K, et al. 2018. Viral diversity of house mice in New York City. mBio. 9(2):e01354–17. 29666290 10.1128/mBio.01354-17 PMC5904411

[CIT0051] Yang S, Liu Z, Wang Y, Li W, Fu X, Lin Y, Shen Q, Wang X, Wang H, Zhang W. 2016. A novel rodent Chapparvovirus in feces of wild rats. Virol J. 13(1):133. 27473724 10.1186/s12985-016-0589-0 PMC4966819

[CIT0052] Yinda CK, Ghogomu SM, Conceição-Neto N, Beller L, Deboutte W, Vanhulle E, Maes P, Van Ranst M, Matthijnssens J. 2018. Cameroonian fruit bats harbor divergent viruses, including rotavirus H, bastroviruses, and picobirnaviruses using an alternative genetic code. Virus Evol. 4(1):vey008. 29644096 10.1093/ve/vey008 PMC5888411

