# Supplementary material for: Carnivore chaphamaparvovirus-1 (CaChPV-1) infection in diarrheic dogs reveals viral endotheliotropism in intestine and lung
Source: Vet Q. 2023 Mar 8;43(1):1–10. doi: 10.1080/01652176.2023.2185696 (PMC10013547; doi:10.1080/01652176.2023.2185696)
Supplement: Supplemental Material [file TVEQ_A_2185696_SM2494.docx]

**Supplementary Table S1** Information of primers used in this study

| Virus | Target gene (^a^) | Primer name | Primer sequence 5’-3’ | Target size (bp) | Reference |
| --- | --- | --- | --- | --- | --- |
| Canine chaphamaparvovirus-1 | NS1  (493-1592) | *ChPV*-1F | TGACTGGTTAGTTCGCTTTC | 1099 | Hu et al., 2020 |
|  |  | *ChPV*-1R | GGTTCTTCCCATACTCCAAT |  |  |
|  | NS1  (1055-2153) | *ChPV*-2F | GCTATTATGATTTAGGAGAACGCTT | 1098 |  |
|  |  | *ChPV*-2R | CTGGTTCGTATCCCGTCGCTA |  |  |
|  | VP1  (2118-3053) | *ChPV*-3F | CTCCTGCACCTCAGTTAGCG | 935 |  |
|  |  | *ChPV*-3R | GCCATACAGCCGATCCAC |  |  |
|  | VP1  (2827-4123) | *ChPV*-4F | TGCACAAGATGATCTATACGAA | 1294 |  |
|  |  | *ChPV*-4R | GGATACACAGGCGCCAGTACAGTA |  |  |
|  | NS1  (1957-2181) | *ChPVF*-p | AGCTCAGTTTGGCCCAGATC | 224 | In this study |
|  |  | *ChPVR*-p | AGAGGGATCGCTGGATCTGT |  |  |
| Canine parvoviruses | VP | VPF | ATGGCACCTCCGGCAAAGA | 2246 | Piewbang et al., 2021 |
|  |  | VPR | TTTCTAGGTGCTAGTTGAG |  |  |
| Canine distemper virus | NP | F768 | AACAGRRATTGCTGAGGACYTAT | 290 | Piewbang et al., 2020 |
|  |  | R1057 | TCCARRRATAACCATGTAYGGTGC |  |  |
| Canine herpesvirus-1 | GB | GBF | ACAGAGTTGATTGATAGAAGAGGTATG | 136 | Piewbang et a., 2017 |
|  |  | GBR | CTGGTGTATTAAACTTTGAAGGCTTTA |  |  |
| Canine adenoviruses | E3 | E3_F | TATTCCAGACTCTTACCAAGAGG | 452-708 | Wardhani et al., 2021 |
|  |  | E3_R | ATAGACAAGGTAGTARTGYTCAG |  |  |
| Canine enteric coronavirus | ORF1 | IN2 | GGGTTGGGACTATCCTAAGTGTGA | 452 | Ksiazek et al., 2003 |
|  |  | IN4 | TAACACACAACICCATCATCA |  |  |
| Canine bocaviruses | VP | CBoV-F | AARAGRAARCTYTATTTTGC | 377-404 | Piewbang et al., 2021 |
|  |  | CBoV-R | TGCCAGTCTTGWGGHGARAA |  |  |
| Canine bufavirus | VP2 | 165F | CTGGTTTAATCCAGCAGACTA | 120 | Martella et al., 2018 |
|  |  | 371R | TGAAGACCAAGGTAGTAGGT |  |  |
| Canine astrovirus | RdRp | Astro-F1 | GARTTYGATTGGRCKCGKTAYGA | 422 | Chu et al., 2008 |
|  |  | Astro-F2 | GARTTYGATTGGRCKAGGTAYGA |  |  |
|  |  | AstroR1 | GGYTTKACCCACATNCCRAA |  |  |
|  |  | Astro-F3 | CGKTAYGATGGKACKATHCC |  |  |
|  |  | Astro-R2 | AGGTAYGATGGKACKATHCC |  |  |
| Canine kobuvirus | RdRp | CaKV-F | CCCTGGAACACCCAAGGCCGCT | 504 | Li et al., 2018 |
|  |  | CaKV-R | TCTGGTTGCCATAGATGTGGTG |  |  |

^a^ nucleotide position according to *Cachavirus-1A* GenBank Accession no. MH983826

GB: glycoprotein B; E = early region; NP = Nucleoprotein; NS = non-structural protein; RdRp = RNA-dependent RNA VP = structural protein
